# Barley Genomics: An Overview

**DOI:** 10.1155/2008/486258

**Published:** 2008-02-13

**Authors:** Nese Sreenivasulu, Andreas Graner, Ulrich Wobus

**Affiliations:** Leibniz-Institut für Pflanzengenetik und Kulturpflanzenforschung (IPK), Corrensstraße 3, 06466 Gatersleben, Germany

## Abstract

Barley (*Hordeum vulgare*), first domesticated in the Near East, is a well-studied crop in terms of genetics, genomics, and breeding and qualifies as a model plant for *Triticeae* research. Recent advances made in barley genomics mainly include the following: (i) rapid accumulation of EST sequence data, (ii) growing number of studies on transcriptome, proteome, and metabolome, (iii) new modeling techniques, (iv) availability of genome-wide knockout collections as well as efficient transformation techniques, and (v) the recently started genome sequencing effort. These developments pave the way for a comprehensive functional analysis and understanding of gene expression networks linked to agronomically important traits. Here, we selectively review important technological developments in barley genomics and related fields and discuss the relevance for understanding genotype-phenotype relationships by using approaches such as genetical genomics and association studies. High-throughput genotyping platforms that have recently become available will allow the construction of high-density genetic maps that will further promote marker-assisted selection as well as physical map construction. Systems biology approaches will further enhance our knowledge and largely increase our abilities to design refined breeding strategies on the basis of detailed molecular physiological knowledge.

## 1. INTRODUCTION

**Figure 1:**
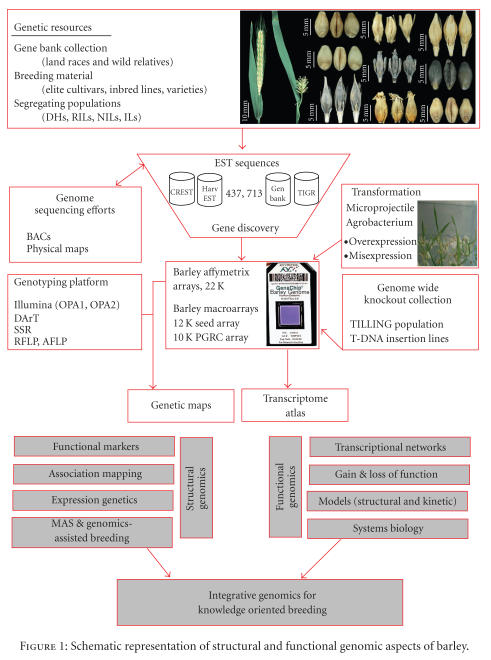
Schematic representation of structural and functional genomic aspects of barley.

In the 21st century, cereals continue to constitute the most important crops with an annual output of 2 billion tons (according to FAO in 2006; http://www.fao.org). In today's worldwide production, barley ranks fourth among cereals and is preferentially used as feed grain, as a raw material for beer production and, to a smaller extent, as food. Initially, barley was domesticated in the fertile crescent of the Neolithic Near East over 10 000 years ago [1]. In the subsequent millennia, farmers continuously adapted local populations to their needs, leading to a great variety of landraces. About 100 years ago, these formed the basis for the development of modern cultivars by cross breeding. During this time, grain yield was more than doubled with an estimated genetic contribution to this increase of about 30–50% [2]. However, to meet the future challenges imposed by a changing environment, to feed a growing world population, and to provide renewable resources to satisfy the soaring demand for energy, genomics-based technologies have to be efficiently implemented to study the genetic basis of plant performance and to isolate agronomically important genes from the genetic diversity present in the gene pool of barley. A broad spectrum of resources has been developed during the last two decades to facilitate the systematic analysis of the barley genome. These include a large number of mapped molecular markers, comprehensive EST collections, BAC libraries, mutant collections, DNA arrays, and enabling technologies such as the large scale production of doubled haploids and efficient transformation protocols. Advances made in barley genomics and recent efforts made towards physical map construction and sequencing of the barley gene space (http://barleygenome.org) will largely contribute to a comprehensive understanding of gene functions in the context of agronomical important phenotypes (refer to Figure 1 and Table 1). Recently, the techniques and methods employed in cereal genomics have been reviewed [3–8]. In this overview, we have tried to summarize progress in structural and functional genomics of barley and put emphasis on important agronomical aspects such as grain yield, seed quality traits, and implications for malting quality improvement.

## 2. BARLEY ESTS, BACS, AND PHYSICAL MAPS—A SPRINGBOARD FOR THE EXPLORATION OF THE GENOME

The seven barley chromosomes represent the basic genome of all Triticeae species. Still, the large genome ( *∼*5500 MB), of which 80% is composed of repetitive DNA is presently not amenable to whole genome sequencing. Therefore, large scale sequencing programs for the development of expressed sequence tags (ESTs) from various cDNA libraries have been initiated. The progress made in the last 5 years resulted in the generation of 437,713 ESTs covering different cDNA libraries from various stages of plant development and tissues challenged with abiotic and biotic stresses (http://www.ncbi.nlm.nih.gov/dbEST/dbEST_summary.html, September 14th 2007 release). Alignment of these ESTs led to the identification of a representative set of 50,453 unigenes with 23,176 tentative consensi and 27,094 singletons (http://compbio.dfci.harvard.edu/tgi/cgi-bin/tgi/gimain.pl?gudb=barley), representing possibly about 75% of all genes in the barley genome. An earlier estimate of the barley gene content based on 110,000 ESTs led to the prediction of around 30 000 unique genes [9]. This number might be an under representation due to the low EST coverage. The same EST data set, which was generated from different tissues covering the plant's life cycle, was analyzed to gain insight into differential gene expression programs in diverse plant tissues by in silico expression studies [9]. In this way, comprehensive analysis of extensive EST resources generated from large genomes provides snap shots of the transcriptome aiding in gene discovery. This also allows identifying coregulated metabolic and regulatory networks [10, 11] and helps to establish high-density molecular maps [12–14] which form the basis for comparative genomic studies, trait mapping, and map-based gene isolation. Thus, in large genome cereal species like barley, EST sequences facilitate a comprehensive overview of gene content and represent a resource to study the evolution and organization of a genome. Regarding the latter, EST-derived information remains limited as it fails to provide, for instance, regulatory information, since promoters and full length sequences are not available.

Physical maps represent an important link to connect the genetic level to the sequence level. Similar to genetic maps, physical maps are available at different levels of resolution. Wheat-barley addition lines are a useful resource to rapidly assign ESTs to an entire chromosome or to a chromosome arm [15]. Using this resource, 1787 genes present on the Barley 1 GeneChip could be assigned to the six different chromosomes of barley (365 genes to 2H, 271 to 3H, 265 to 4H, 323 to5H, 194 to 6H, and 369 to 7H) [16]. At a higher resolution, a physical map of all the seven barley chromosomes has been prepared by mapping DNA markers derived from both genomic as well as gene-based sequences relative to the translocation breakpoints of individual chromosomes that had been isolated using microdissection techniques [17]. The resulting map is of particular value, as it can be directly aligned to the genetic map of barley by common markers and thus allows for the estimation of the ratio between genetic and physical distances. An alternative approach has been described by Masoudi-Nejad et al. [18]. Here the presence of a wheat gametocidal chromosome in a wheat barley addition line was exploited to select 90 progeny lines that carried differently sized fragments of barley chromosome 7H. These were subsequently used to determine the physical order and distance of markers located on barley chromosome 7H.

During the past several years, core public resources have been established by generating “bacterial artificial chromosome” (BAC) libraries from different barley cultivars: “Morex” ([19]; 313,344 clones), “Cebada Capa” ([20]; 177,000 clones) and “Haruno Nijo” (http://www.intl-pag.org/10/abstracts/PAGX_P393.html). Based on fluorescence in situ hybridization (FISH) techniques karyotype landmarks were derived for barley, which could be used in future to place the BAC clones onto the physical map [21]. This map shows that the genetic linkage maps are well covered with markers among all chromosomes. At the same time, the physical maps reveal large areas of the barley genome that have yet to be mapped. These unmapped areas mainly consist of heterochromatin and show very low recombination rates [17]. In accordance with these findings, there is increasing evidence that genes are not randomly distributed across the barley genome but confined to a gene space, which mainly covers the distal parts of the chromosomes. Experimental evidence for the existence of a gene space has been gained from screening a barley BAC library with EST-derived probes, which showed a significant nonrandom distribution across the BAC clones [22]. More direct evidence has been reached on the sequence level for barley and other Triticeae species. Although up to now only a limited amount of sequence data is available, the average density of annotated genes is much higher than that expected for a random distribution across the genome. The disproportionate gene number found is probably due to the preferential selection of gene containing BACs for sequence analysis. Within single BACs, there is considerable variation ranging, in case of barley, from 1 gene in 12 kb up to 1 gene in 220 kb (for review see [23]). Thus even the gene space itself seems to be characterized by a highly variable distribution of genes against the backdrop of noncoding, mainly repetitive DNA.

The existence of a gene space also opens up new opportunities to focus analyses on gene-rich regions only. Recently, international efforts have been gearing up to utilize the extensive barley EST resources for BAC anchoring and genetic mapping. An elegant approach of screening of the Morex BAC library using EST-derived, pooled “overgo” probes [24] resulted in the identification of gene containing BACs. Upon fingerprinting of a subset of 21 161 clones, 2262 contigs could be assembled covering approximately 9.4% of the barley genome. Furthermore, a database has been set up to search screening results of BAC libraries as well as to provide an integrative view of data from the existing barley genetic and physical maps (http://www.genome.clemson.edu). The identified BAC-based gene-rich regions of the genome have been selected as a genomic reference from cultivar Morex to initiate sequencing of all gene-containing regions of the barley genome by an international effort coordinated through the International Barley Sequencing Consortium (IBSC, http://barleygenome.org).

## 3. A BARLEY TRANSCRIPTOME ATLAS

Despite the lack of a barley genome sequence, functional genomics efforts have been initiated by taking advantage of the available EST sequence information generated by multinational coordinated efforts (see above). As a first step, efforts were made to derive functional assignments of the available barley unigene set by annotation transfer from homologous sequences relying on the available plant whole genome sequences and by identifying common motifs from Interpro. As a result, several ontology structures such as MIPS [9] and MAPMAN functional categories (N. Sreenivasulu, unpublished data; http://mapman.mpimp-golm.mpg.de/index.shtml) were developed. Such computational methods also yielded putative regulatory networks as well as metabolic pathway interaction networks, but still about half of the genes have to be classified as “unknown.”

The available barley EST unigene resources played a profound role in developing several platforms for transcriptome analysis including cDNA-based macroarrays [11, 25], microarrays [26], and oligonucleotide-based affymetrix arrays [10, 27]. Other profiling techniques used in barley include cDNA-AFLP [28], SAGE (Serial Analysis of Gene Expression) [29, 30], and iGentifier. The latter method combines elements of tag sequencing such as SAGE and fragment display [31]. By successfully applying these techniques, barley transcriptome data have been collected from grain development [11, 25, 32], grain germination [33, 34], at least 15 different tissues/organs covering different growth stages [10], and abiotic [26, 35–37] as well as biotic stress responses [38, 39]. The new insights gained from transcriptome analysis of host-pathogen studies have lately been reviewed by Wise et al. [40]. These large scale gene expression data sets serve as baseline experiments to generate a barley transcriptome atlas. Also, an online Plant Expression Database (PLEXdb), previously known as BarleyBase (http://www.plexdb.org/plex.php?database=Barley) has been created to store, visualize, and statistically analyze Barley 1 GeneChip data [41].

While transcriptomics have brought about substantial progress in elucidating biochemical pathways of barley seed metabolism (see reviews [5, 42]), very recent findings shed light on the interplay of many cellular and metabolic events that are coordinated by a complex regulatory network during barley seed development [10, 11, 25]. Studying expression data of nearly 12 000 seed-expressed genes revealed, for instance, the participation of tissue-specific signaling networks controlling ABA-mediated starch accumulation (via SNF1 kinase and a set of transcription factors) in the endosperm and participation of ABA-responsive genes in establishing embryo desiccation tolerance [11].* *CpG methylation found in the promoters of prolamin box-binding factor and B-hordein genes suppresses transcript levels during the prestorage until the intermediate phase of grain development. This process coincides with the coexpression of methyltransferases, core histones and DNA-unwinding ATPases [43]. Thus storage protein gene expression may be regulated by CpG methylation. Using a *lys 3a* mutant, it has been shown that demethylation of the B-hordein promoter does not occur in the mutant, hence transcripts encoding storage proteins such as B-hordeins and C-hordeins are almost absent in the developing endosperm of this mutant [44]. Transcriptome profiling of barley embryos using the 22K affymetrix Barley 1 GeneChip revealed activation of developmentally distinct defense related gene sets including coregulated phenylpropanoid and phytoalexin related genes around 20 days after flowering (DAF), followed by upregulation of antioxidant and pathogen related gene sets around 37 DAF [45]. The knowledge obtained on metabolic processes of seed quality traits could eventually be used to develop superior varieties by genetic engineering or by marker-assisted selection in conventional breeding programs.

Transcriptome analysis has also been carried out during barley grain germination at tissue-specific levels [10, 46]. Using cDNA array technology gene expression was analyzed in germinating seed samples, collected from ten different barley genotypes showing differential malting response [46]. Based on six different malting quality parameters related to hydrolytic events connected to protein, starch, and cell wall degradation 19 candidate genes were identified, whose transcript abundance showed a significant correlation with some of the malting quality parameters. White et al. [30] analyzed seven different SAGE libraries derived from malted grains and identified 100 most abundant transcripts showing differential responses during eight different time points during malting. These transcripts are related to stress and defense response, hydrolytic processes and translational events. The list of candidate genes identified in the two studies [30, 46] was further validated by a genetical genomics approach in which gene expression studies were conducted with populations segregating for malting traits [34, 47].

## 4. FUNCTIONAL GENOMICS APPROACHES IN BARLEY

A major aim of functional genomic studies is to understand the metabolic and regulatory networks within the structural and functional context of cells, tissues, and organs often changing with time. Hence in this review, we update the functional genomic resources available (Table 1) to study gene functions in barley using reverse genetics approaches and highlight the initial success achieved through genetic engineering based on the manipulation of individual genes.

### 4.1. Reverse genetics

To determine gene-function relationships, large scale genome-wide reverse genetics approaches have been developed in barley (see [48] for review) which includes both nontransgenic technology platforms such as TILLING (targeting induced local lesions in genomes) [49] and insertional mutagenesis systems based on transgenic technology [50–54]. Thus, the Scottish Crop Research Institute generated a large M_2_ TILLING population in the barley cultivar “Optic” with leaf material and seeds from 20 000 plants freeze dried and archived [49]. EMS induced mutations were scored at various growth stages under different conditions and documented [49, 55]. Mutant phenotypes, candidate genes, and observed DNA sequence variations can be queried in an SCRI mutant database (http://germinate.scri.ac.uk/barley/mutants/index.php?option=com_wrapper&Itemid=35). In a more recent attempt, IPK developed a TILLING population of 10 000 M2 plants in the cultivar ‘Barke” (N. Stein, personnel communication). Similarly, a collection of 5000 M3 mutants of the cultivar “Morex” is provided by the University of Bologna (http://www.intl-pag.org/13/abstracts/PAG13_P081.html).

To aid functional gene analysis, insertional mutagenesis approaches were followed in barley during the last decade (i) to create loss-of-function mutations by the insertion of transposable elements into a gene of interest [50–53] and (ii) use activation tagging (the random genomic insertion of either promoter or enhancer sequences) to generate dominant gain-of-function mutations [54, 56]. Insertion lines have been generated by creating transgenic plants carrying *Ac* and *Ds* elements, and crossed them to induce *Ds* transposition [50–52]. *Ds* elements were preferentially found in genic regions and exhibited a high-remobilization frequency [52, 53]. Such *Ds* launch pads, represented by barley lines with each harboring a single copy *DS* insertion at a well-defined position in the genome, will be valuable for future targeted gene tagging. Similarly, dominant overexpression phenotypes [54, 56] will help to study gene functions in the large barley genome where loss-of-function mutations often may not cause phenotypes because of gene redundancy.

### 4.2. Transgenic barley and its potential applications

In order to functionally characterize candidate genes identified in functional genomic studies, it was mandatory to establish a stable and efficient genetic transformation technique in barley. In contrast to the biolistic gene transfer technique [57], a more efficient *Agrobacterium* mediated barley genetic transformation method based on immature embryos was developed in spring barley [58]. In a recent attempt to further improve this technology, Kumlehn et al. [59] developed a transformation method for winter barley based upon the infection with *Agrobacterium* of androgenic pollen cultures. By this approach, homozygous double haploid plants could be immediately obtained at high frequency through chromosome doubling.

During the last decade, systematic efforts were made for genetic engineering of barley to improve seed quality traits including those related to malting (reviewed in [60]). Malting improvement has been addressed by altering the expression of hydrolytic enzymes related to the degradation of storage products such as starch (*α* and *ß*-amylases, [61, 62]) and cell wall components. In another approach, several enzymes such as xylanase, glucanase, endo-, and exoprotease were over expressed in transgenic barley grains and preferably the enzyme mix necessary for malting process are provided by transgenic seeds [63].

Protein engineering has been used to produce thermostable 1, 3; 1, 4*ß*-glucanases in transgenic barley grains [64–66]. Such grains can be used to enhance the feed quality of barley for poultry [67, 68]. In a similar approach, a hybrid cellulase gene driven by the endosperm specific rice GluB-1 promoter was expressed and produced the enzyme up to 1.5% of total grain protein [69]. In addition, functions of key genes involved in determining seed quality traits related to storage product accumulation were tested. For instance, antisense downregulation of limit dextrinase inhibitor showed reduced amylose over amylopectin levels and eventually reduced total starch [70]. Also overexpression of wheat thioredoxin *h* in the endosperm of transgenic barley grain leads to increased activity of the starch debranching enzyme limit dextrinase [71, 72]. Further, a powerful approach of antisense oligodeoxynucleotide inhibition has been used to reveal sugar signaling networks. Short stretches of 12–25 nucleotide long single-strand sequences have been delivered to barley leaf cells to block the effect of SUSIBA2, a key transcriptional activator involved in plant sugar signaling [73]. Recently, this approach has been successfully implemented to deliver antisense oligodeoxynucleotides to barley seed endosperm to suppress sugar related signaling genes [74]. HvGAMYB, a transcription factor initially identified in aleurone and shown to be upregulated by gibberellin, has been shown to be expressed also in barley anthers. The overexpressing HvGAMYB transgenic lines show reduced anther size with a male sterility phenotype [75]. Our laboratory has recently characterized a new protein called Jekyll, which is preferentially expressed in barley grain nucellar projection tissue [76]. Its downregulation decelerates autolysis of nurse tissue. As a result, proliferation of endosperm nuclei is impaired and less starch is finally accumulated in the endosperm [77].

### 4.3. Towards systems biology

With respect to applied aspects in crop plants, a comprehensive knowledge of cellular and functional complexity as related to key agronomic traits could be revealed using a systems biology approach. With this in mind, a number of tools and databases were developed at our institute (Leibniz Institute of Plant Genetics and Crop Plant Research/IPK) to store, analyze, and display the data derived from multiparallel-OMICs profiling studies at transcript, metabolite, and protein/enzyme level with the aim to eventually gain insight into the organization of function-related networks in barley [78, 79]. These include CR-EST [78] (it provides access to clustering and annotation data of IPK EST projects), Meta-All [79], and MetaCrop [80] (they allow to access curated metabolic pathway information and kinetic reactions of crop plants), VANTED [81] (for visualization and analysis of metabolic and regulatory networks), HiT-MDS [32] (for screening of coexpressed genes and validation of cluster centroids) as well as barley MapMan and PageMan [http://mapman.mpimp-golm.mpg.de; to index and visualize overrepresented functional categories and detailed metabolic pathway charts from throughput transcriptome data]. With the focus of using the “developing seed” as model for systems biology studies, we investigated transcriptional and metabolic networks during grain development [11, 25, 82], developed 3D models of the developing barley grain [83], implemented magnetic resonance-based techniques to establish 4D models as a framework to store different sets of data in their spatiotemporal context [84], visualized the spatial distribution of specific biochemical compounds by noninvasive NMR-based imaging methods [85] and established kinetic models of primary metabolism ([86] and E. Grafahrend-Belau and B. Junker, unpublished data) as already worked out for potato [87]. In addition, a proteomic platform has been successfully established to study barley grain development [88, 89]. The emerging model (largely qualitative) explaining how the barley grain develops and functions has to be further validated especially by the creation and analysis of different lines of transgenic plants with perturbations at putative key metabolic and/or regulatory sites (see Figure 1).

## 5. FUNCTIONAL MOLECULAR MARKERS AND THEIR POTENTIAL APPLICATIONS IN THE AREA OF APPLIED GENOMICS

### 5.1. Marker development and marker-assisted selection (MAS)

Almost two decades ago, RFLP markers were employed to develop the first comprehensive molecular marker maps in barley [90–92]. Using those RFLP maps, a series of agronomic traits and characters including many quality traits and resistance against several diseases have been mapped (for review see [93, 94]). Later, the availability of large numbers of ESTs facilitated the systematic development of functional markers, for example, by extracting ESTs containing simple sequence repeat (SSR) motifs using appropriate software tools [95]. Although EST-based SSR markers have been shown to be less polymorphic than their genomic counterparts, this drawback is more than compensated for by the ease of their development. Also, the availability of ESTs from multiple-genotypes/cultivars of barley provides the possibility to identify sequence polymorphisms (mainly single-nucleotide polymorphisms and small InDels) in the corresponding EST alignments. These in turn can be exploited for the development of markers [96, 97]. Kota et al. [98] developed the computer algorithm SNiPping for discovery of functional markers through browsing EST assemblies in barley. Also an SNP2CAPS program has been published to facilitate the computational conversion of SNP markers into CAPS markers [99]. Information generated from the diverse mapping projects was further enhanced by the development of consensus maps [14, 100–102]. These provide integrative genetic information by featuring high marker densities. Although the gel-based genotyping platforms offer the best quality marker systems, their low throughput encouraged researchers to explore high-throughput technologies that can simultaneously assay thousands of markers based on single nucleotide polymorphisms (SNP). Most recently, genome-wide scans using SNP-based genotyping platforms such as Illumina GoldenGate BeadArrays [103] and the diversity arrays technology (DArT), which do not require any sequence information [104] have been successfully established in barley. Although DArTs are not systematically interrogating expressed sequences, the choice of appropriate enzymes facilitates their enriched representation. Based on DArT technology, a high-density consensus map has recently been established [105]. A number of recent studies also reported the use of the affymetrix Barley 1 GeneChip [27] for identifying single-feature polymorphisms (SFPs), which cover not only SNPs but also indels and polymorphisms generated due to alternative splicing and polyadenylation [34, 106].

An important application of the above discussed functional markers is marker-assisted selection (MAS). MAS is based on linking the DNA polymorphisms revealed by marker analysis with agronomical traits allowing for their rapid selection in routine breeding programs. MAS can be performed already at juvenile growth stages and before flowering, and thus provides breeders with the opportunity to implement faster back-crossing strategies and allele enrichment in complex crosses, which eventually reduces the time and costs required for the development of improved varieties. Despite its inherent advantages, the application of MAS in barley up to now has mainly been restricted to monogenic traits such as disease resistances. Here, one of the most widespread examples is the marker assisted selection of the *rym4* gene giving resistance to the barley yellow mosaic virus complex. For this gene, several closely linked and easily scorable markers have been developed [107, 108]. More recently, cloning of the gene facilitated the exploitation of functional polymorphisms within the coding region of the resistance gene to differentiate between alleles [109]. Using MAS, several genes providing full resistance could be readily combined in complex crosses without time consuming progeny tests in the greenhouse or in the field (e.g., [110, 111]).

MAS for quantitative traits suffers from two major limitations. (i) Compared to monogenic traits, quantitative traits are characterized by lower heritabilities impairing their accurate scoring and entailing a less accurately defined genetic position of the corresponding quantitative trait locus (QTL). As a result, large chromosomal fragment needs to be selected for, resulting in the meiotic transfer of many potentially undesired genes. Meiotic purification of a QTL into a “mendelian” locus, showing monogenic inheritance, provides a solution to this problem. The feasibility of downtracking a QTL to a single gene has been initially demonstrated in tomato and requires the stepwise size reduction of a QTL fragment and its conversion into a near isogenic line by repeated backcrossing (for review see [112]). In barley, this approach has been successfully employed to isolate the bot1 gene underlying a major QTL conferring boron tolerance [113]. (ii) Many of QTL alleles escape detection, when transferred into a different genetic background. The reasons for the “disappearance of QTLs” include epistatic interactions, QTL x environment effects, the allelic states of the parental lines or the small contribution of a single QTL to the overall variance. As a result, only few common QTLs were detected, when the results of mapping studies that were performed in different crosses were compared [114].

Although the number of successful examples for applying MAS in barley breeding is still rather limited (see reviews by [114, 115], the recent implementation of high-throughput genotyping platforms (Illumina, DArT, and SFP identification by using Barley 1 GeneChip affymetrix array) in barley will significantly increase the identification of marker trait associations, and the subsequent identification of potential candidate genes. Finally, this will allow to treat QTLs as monogenic traits and thus spur their marker assisted manipulation in breeding programs. In combination with a wide range of mapping populations developed for specific agronomic traits, this comprehensive resource of markers now allows the identification of polymorphisms in functionally defined sequences [12, 34, 105, 106]. Functional markers will also be useful for (i) association studies based on linkage disequilibrium, (ii) detection of *cis* and *trans*-acting regulators either based on genetical genomics studies using well-defined mapping populations or by investigating allelic imbalance [116], (iii) identification of alleles influencing agronomically important traits using TILLING/EcoTilling approaches (EcoTilling is a means to determine the extent of natural variation in selected genes), and (iv) genomics-assisted breeding (see Figure 1).

### 5.2. Linkage disequilibrium-based association studies

Linkage disequilibrium is the nonrandom distribution of alleles in a sample population and forms the basis for the construction of genetic maps and the localization of genetic loci for a variety of traits. The principles leading to LD apply to both biparental mapping populations (F2, RILs, etc.) and natural populations. Therefore, LD mapping is the method of choice for genetic analysis in organisms like humans and animals, where experimental populations are either not available or difficult to establish [117].

Because of its inherent advantages, LD mapping approaches are increasingly being applied for plant species, in particular maize. Due to the outbreeding character of this species, LD extends only over a few kb and thus leads to a high-genetic resolution, up to the level of individual candidate genes that can be associated with a given trait (see recent reviews [118, 119]). The use of association genetic analyses in inbreeding species such as barley has been limited so far. However, recent studies have shown that LD extends over much longer genetic distances in barley than in maize. A European germplasm collection of 146 two-rowed spring barley cultivars was used to carry out LD mapping of yield traits using 236 AFLP markers [120]. Associated markers were identified that are located in similar regions where QTLs for yield had been found in barley [93, 121, 122]. A systematic survey of 953 gene bank accessions representing a broad spectrum of the genetic diversity in barley genetic resources revealed that LD extends up to 50 cM but is highly dependent on population structure [120, 123]. On the one hand, the high level of LD in barley is due to the inbreeding mating type of this species; on the other hand, the selection of germplasm plays an important role. Analysis of a germplasm collection of European cultivars, land races, and wild barley accession from the Fertile Crescent region provided hints that the level of LD decreases from cultivars to landraces to wild barley [124]. Similarly, Morrell et al. [125] reported low levels of LD in wild barley by examining LD within and between 18 genes from 25 accessions. Local differences in LD have been observed at the grain hardness locus comprising four closely linked genes (*hinb*, *hina*, GSP, PG2). Here, a high level of LD was observed in the intergenic region between *hinb-1* and *hina* probably due to transposable elements present in this region, which influence the local recombination rate [114]. By assaying 1524 genome-wide SNPs in elite northwest European barley using the Illumina GoldenGate BeadArray platform Rostoks et al. [103] concluded that whole-genome association scans can be exploited for trait mapping in barley. This was further exemplified by the identification of a marker that showed an association with the winter habit and which could be tracked to a cluster of CBF (C-repeat/DRE-binding factor) gene homologs. In a recent whole genome LD-mapping approach, Steffenson et al. [126] used 318 wild barley accessions to perform association mapping studies using DArT markers to identify rust resistance genes. In addition, LD analysis has been performed based on haplotypes derived from 131 accessions by covering 83 SNPs within 132 kb around the gene HveIF4E, which confers resistance to barley yellow mosaic virus. The authors identified three haplogroups discriminating between the alleles *rym4* and *rym5* [127]. Taken together, the above mentioned association studies provide starting points for a more systematic analysis of agronomic traits. These may be selected from the vast ex situ gene bank collections available for this crop. Alone at the IPK gene bank some 20 000 different barley accessions represent an ample cross section of the genetic diversity present in this species. However, in order to fully exploit the potential of LD-based association analysis in this species, populations have to be carefully selected to minimize the confounding effects of population structure. This is particularly evident in modern barley germplasm, which is frequently structured into spring and winter as well as 2-rowed and 6-rowed types, forming distinct subpopulations (e.g., [95]). If these effects are not adequately accounted for during association analysis, the risk of detecting spurious associations increases.

### 5.3. Genetical genomics studies

The genetical genomics strategy was first outlined by Jansen and Nap [128]. It combines gene expression studies with genetic linkage analysis. Differentially expressed genes (but also proteins and metabolites) involved in metabolic and regulatory pathways and identified by high-throughput technologies are treated as phenotypes, and genetic variants that influence gene expression are identified in genetically related lines. This strategy has been successfully applied also in plant systems, and relevant data were reviewed elsewhere [128–130]. Here, we will focus on the latest development in expression QTL (eQTL) mapping in barley. Using the Barley 1 GeneChip affymetrix array SFP genotyping has been performed in 35 recombinant lines of a Steptoe × Morex doubled-haploid population, enabling eQTL studies [34]. Using a high-throughput SFP genotyping platform, genome-wide linkage analysis has been performed based on 22 000 transcript data collected from 139 DH lines (Steptoe × Morex). The most significant eQTLs derived from germinating barley grain are linked to *cis* regulation [47]. Using the same mapping population, a serine carboxypeptidase 1 eQTL has been mapped on chromosome 3H to the same region where a QTL for the malting quality trait “diastatic power” has been mapped [131]. In another study, instead of a segregating population a set of 47 BC3 DH introgression lines was employed (wild barley [*H. spontaneum*] is introgressed in the genetic background of the elite line “Brenda” [*H. vulgare*]) in order to understand gene expression networks controlling seed traits. Initially, this BC3 DH population was used to identify QTLs for yield and yield components [132]. In further experiments, expression data from nearly 12 000 genes interrogated by using a barley seed specific array were used to calculate eQTLs (C. Pietsch et al., unpublished). Although such initial studies provide evidence that genetical genomics is a promising concept which assists to expose gene-trait relationships, an extensive exploration of the technology needs the full barley genome sequence and improved high-throughput genotyping information.

## 6. OUTLOOOK

In recent years, we experienced a dramatic development of new tools and technologies for genome research and a concomitantly dramatic increase in data leading to a much improved and advanced knowledge base. Barley research gained a lot of momentum from this development but the nonavailability of a whole genome sequence is still a serious limitation. However, due to consortial efforts (see above) and the rapidly developing sequencing technologies that are relevant for even complex genomes like that of barley [133] this limitation will be largely overcome, hopefully within the next five years. High-throughput transcriptome analysis techniques have already provided numerous new insights in transcriptional networks. They will, together with rapidly improving protein and metabolite profiling techniques and in combination with new genetic analysis concepts such as genetical genomics and association genetics, improve our knowledge on the relationship between the genetic and the phenotypic architecture of agronomic traits and thus create a basis for knowledge-based molecular breeding [134]. As a next step systems biology approaches are emerging, which attempt to model complex cellular or organismic functions in response to changing internal and external factors [135]. Until now molecular markers have had limited success in barley breeding programs, but due to recent advancement of barley genomics a stronger impact on breeding strategies is expected. For instance, marker technologies together with double haploid production have almost halved the time of variety development in Australian wheat and barley breeding programs [136]. However, new whole-genome breeding strategies have to be developed to make full use of the ever increasing knowledge about crop plant genomes and their behavior.

## Figures and Tables

**Table 1:** Barley genomic resources.

Databases	Website URL	Application
Barley Genetic Stocks	http://ace.untamo.net/cgi-bin/ace/searches/basic	Provides information on the morphological & genetic background of barley mutants and contains information on 736 barley translocation and duplication lines.
US Barley Germplasm	http://barleyworld.org/northamericanbarley/germplasm.php	Contains information on barley recombinant chromosome substitution lines and North American barley mapping populations.
EBDB	http://pgrc-35.ipk-gatersleben.de/portal/page/portal/PG_BICGH/P_BICGH/P_BICGH_RESOURCES/P_BICGHI_RESEBDB	The European Barley Database (EBDB) contains passport and evaluation data of 155,000 barley accessions including the international barley core collection.
ICARDA Barley varieties	http://www.icarda.cgiar.org/Crops_Varieties.htm#Barley	Provides an index of barley variety releases from ICARDA, 1977–2005.
Barley TILLING	http://www.scri.ac.uk/research/genetics/BarleyTILLING	A reverse genetics platform, which can be screened for 8,600 barley (cultivar “Optic”) EMS mutagenized lines.
CR-EST	http://pgrc.ipk-gatersleben.de/cr-est/index.php	Barley EST database containing sequences, functional annotation and clustering information of more than 232,000 ESTs.
HarvEST	http://harvest.ucr.edu	Barley EST database containing unigene sequences and the oligo design of Barley1 Affymetrix array. It also includes a 1000 barley SNP loci genetic map showing syntenic information with rice.
HvGI	http://compbio.dfci.harvard.edu/tgi/cgi-bin/tgi/gimain.pl?gudb=barley	This *Hordeum vulgare* Gene Index provides functional annotation information, 70-mer oligo predictions and in silico gene expression data for 50,000 unigenes.
NCBI Barley genome view	http://www.ncbi.nlm.nih.gov/mapview/map_search.cgi?taxid=4513	Provides an overview about the available genomic and genomic survey sequences (GSS) of barley.
IBSC	http://barleygenome.org	Activities of the International Barley Genome Sequencing Consortium (IBSC) are highlighted.
Barley genome	http://phymap.ucdavis.edu:8080/barley	Barley physical mapping database and available BAC clones together with the accompaning ESTs.
Barley physical map	http://pgrc.ipk-gatersleben.de/kuenzel/barleymap.html	Barley translocation breakpoints integrated into the Igri/Franka-derived RFLP linkage map.
Barley genomics	http://barleygenomics.wsu.edu	Contains information about barley molecular markers, genetic maps, BACs and mutants.
Barley DB	http://ukcrop.net/perl/ace/search/BarleyDB	Contains information about barley germ plasm, molecular markers, genetic maps and BACs.
Gramene	http://www.gramene.org	Provides an overview of comparative maps of cereals including available updated molecular markers and maps of barley.
GrainGenes	http://wheat.pw.usda.gov/GG2/index.shtml	Triticeae database provides an overview about available maps, genetic markers, QTLs and gene expression data.
Barley dbEST SSRs	http://www.genome.clemson.edu/projects/barley/ssr.dbest.html	15,182 barley simple sequence repeats (SSR) were predicted using the available 328,724 dbEST dataset.
Barley SNP database	http://bioinf.scri.ac.uk/barley_snpdb	Barley SNP linkage map.
Barley RFLP database	http://pgrc.ipk-gatersleben.de/rflp/rflp.html	Contains data of mapped barley RFLP-markers from IPK.
Barley DArT	http://www.triticarte.com/content/barley_diversity_analysis.html	High density consensus map of barley DArT markers linking to existing SSR, RFLP and STS loci.
BarleyBase	http://www.plexdb.org/plex.php?database=Barley	An online dataset for storing and visualizing gene expression data of the Barley 1 GeneChip Affymetrix array.
BDC-GED	http://pgrc.ipk-gatersleben.de/seeds	Contains barley developing caryopses gene expression data.

## References

[1] Salamini F, Özkan H, Brandolini A, Schäfer-Pregl R, Martin W (2002). Genetics and geography of wild cereal domestication in the near east. *Nature Reviews Genetics*.

[2] Schuster WH (1997). Welchen Beitrag leistet die Pflanzenzüchtung zurLeistungssteigerung von Kulturpflanzenarten. *Pflanzenbauwissenschaften*.

[3] Rafalski A (2002). Applications of single nucleotide polymorphisms in crop genetics. *Current Opinion in Plant Biology*.

[4] Varshney RK, Hoisington DA, Tyagi AK (2006). Advances in cereal genomics and applications in crop breeding. *Trends in Biotechnology*.

[5] Wobus U, Sreenivasulu N, Freitag J (2006). Genomics approaches for the improvement of cereals. *European Training and Networking Activity, Plant Genomics and Bioinformatics Expression Micro Arrays and Beyond—A Course Book*.

[6] Bagge M, Xia X, Lübberstedt T (2007). Functional markers in wheat. *Current Opinion in Plant Biology*.

[7] Milligan AS, Lopato S, Langridge P, Gupta KP, Varshney RK (2004). Functional genomics of seed development in cereals. *Cereal Genomics*.

[8] Varshney RK, Langridge P, Graner A (2007). Application of genomics to molecular breeding of wheat and barley. *Advances of Genetics*.

[9] Zhang H, Sreenivasulu N, Weschke W (2004). Large-scale analysis of the barley transcriptome based on expressed sequence tags. *The Plant Journal*.

[10] Druka A, Muehlbauer G, Druka I (2006). An atlas of gene expression from seed to seed through barley development. *Functional & Integrative Genomics*.

[11] Sreenivasulu N, Radchuk V, Strickert M, Miersch O, Weschke W, Wobus U (2006). Gene expression patterns reveal tissue-specific signaling networks controlling programmed cell death and ABA- regulated maturation in developing barley seeds. *The Plant Journal*.

[12] Rostoks N, Mudie S, Cardle L (2005). Genome-wide SNP discovery and linkage analysis in barley based on genes responsive to abiotic stress. *Molecular Genetics and Genomics*.

[13] Hearnden PR, Eckermann PJ, McMichael GL, Hayden MJ, Eglinton JK, Chalmers KJ (2007). A genetic map of 1,000 SSR and DArT markers in a wide barley cross. *Theoretical and Applied Genetics*.

[14] Stein N, Prasad M, Scholz U (2007). A 1,000-loci transcript map of the barley genome: new anchoring points for integrative grass genomics. *Theoretical and Applied Genetics*.

[15] Islam AKMR, Shepherd KW, Sparrow DHB (1981). Isolation and characterization of euplasmic wheat-barley chromosome addition lines. *Heredity*.

[16] Cho S, Garvin DF, Muehlbauer GJ (2006). Transcriptome analysis and physical mapping of barley genes in wheat-barley chromosome addition lines. *Genetics*.

[17] Künzel G, Korzun L, Meister A (2000). Cytologically integrated physical restriction fragment length polymorphism maps for the barley genome based on translocation breakpoints. *Genetics*.

[18] Masoudi-Nejad A, Nasuda S, Bihoreau M-T, Waugh R, Endo TR (2005). An alternative to radiation hybrid mapping for large-scale genome analysis in barley. *Molecular Genetics and Genomics*.

[19] Yu Y, Tomkins JP, Waugh R (2000). A bacterial artificial chromosome library for barley (*Hordeum vulgare* L.) and the identification of clones containing putative resistance genes. *Theoretical and Applied Genetics*.

[20] Isidore E, Scherrer B, Bellec A (2005). Direct targeting and rapid isolation of BAC clones spanning a defined chromosome region. *Functional & Integrative Genomics*.

[21] Stephens JL, Brown SE, Lapitan NLV, Knudson DL (2004). Physical mapping of barley genes using an ultrasensitive fluorescence in situ hybridization technique. *Genome*.

[22] Varshney RK, Grosse I, Hähnel U (2006). Genetic mapping and BAC assignment of EST-derived SSR markers shows non-uniform distribution of genes in the barley genome. *Theoretical and Applied Genetics*.

[23] Stein N (2007). Triticeae genomics: advances in sequence analysis of large genome cereal crops. *Chromosome Research*.

[24] Madishetty K, Condamine P, Svensson JT, Rodriguez E, Close TJ (2007). An improved method to identify BAC clones using pooled overgos. *Nucleic Acids Research*.

[25] Sreenivasulu N, Altschmied L, Radchuk V, Gubatz S, Wobus U, Weschke W (2004). Transcript profiles and deduced changes of metabolic pathways in maternal and filial tissues of developing barley grains. *The Plant Journal*.

[26] Ozturk ZN, Talamé V, Deyholos M (2002). Monitoring large-scale changes in transcript abundance in drought- and salt-stressed barley. *Plant Molecular Biology*.

[27] Close TJ, Wanamaker SI, Caldo RA (2004). A new resource for cereal genomics: 22K barley genechip comes of age. *Plant Physiology*.

[28] Leymarie J, Bruneaux E, Gibot-Leclerc S, Corbineau F (2007). Identification of transcripts potentially involved in barley seed germination and dormancy using cDNA-AFLP. *Journal of Experimental Botany*.

[29] Ibrahim AFM, Hedley PE, Cardle L (2005). A comparative analysis of transcript abundance using SAGE and Affymetrix arrays. *Functional & Integrative Genomics*.

[30] White J, Pacey-Miller T, Crawford A (2006). Abundant transcripts of malting barley identified by serial analysis of gene expression (SAGE). *Plant Biotechnology Journal*.

[31] Fischer A, Lenhard A, Tronecker H (2007). *iGentifier*: indexing and large-scale profiling of unknown transcriptomes. *Nucleic Acids Research*.

[32] Strickert M, Sreenivasulu N, Usadel B, Seiffert U (2007). Correlation-maximizing surrogate gene space for visual mining of gene expression patterns in developing barley endosperm tissue. *BMC Bioinformatics*.

[33] Potokina E, Sreenivasulu N, Altschmied L, Michalek W, Graner A (2002). Differential gene expression during seed germination in barley (*Hordeum vulgare* L.). *Functional & Integrative Genomics*.

[34] Luo ZW, Potokina E, Druka A, Wise R, Waugh R, Kearsey MJ (2007). SFP genotyping from affymetrix arrays is robust but largely detects *cis*-acting expression regulators. *Genetics*.

[35] Ueda A, Kathiresan A, Bennett J, Takabe T (2006). Comparative transcriptome analyses of barley and rice under salt stress. *Theoretical and Applied Genetics*.

[36] Talamé V, Ozturk NZ, Bohnert HJ, Tuberosa R (2007). Barley transcript profiles under dehydration shock and drought stress treatments: a comparative analysis. *Journal of Experimental Botany*.

[37] Walia H, Wilson C, Condamine P, Liu X, Ismail AM, Close TJ (2007). Large-scale expression profiling and physiological characterization of jasmonic acid-mediated adaptation of barley to salinity stress. *Plant, Cell & Environment*.

[38] Caldo RA, Nettleton D, Wise RP (2004). Interaction-dependent gene expression in *Mla*-specified response to barley powdery mildew. *Plant Cell*.

[39] Gjetting T, Hagedorn PH, Schweizer P, Thordal-Christensen H, Carver TLW, Lyngkjær MF (2007). Single-cell transcript profiling of barley attacked by the powdery mildew fungus. *Molecular Plant-Microbe Interactions*.

[40] Wise RP, Moscou MJ, Bogdanove AJ, Whitham SA (2007). Transcript profiling in host-pathogen interactions. *Annual Review of Phytopathology*.

[41] Shen L, Gong J, Caldo RA (2005). BarleyBase—an expression profiling database for plant genomics. *Nucleic Acids Research*.

[42] Wobus U, Sreenivasulu N, Borisjuk L (2005). Molecular physiology and genomics of developing barley grains. *Recent Research Developments in Plant Molecular Biology*.

[43] Radchuk VV, Sreenivasulu N, Radchuk RI, Wobus U, Weschke W (2005). The methylation cycle and its possible functions in barley endosperm development. *Plant Molecular Biology*.

[44] Sørensen MB (1992). Methylation of B-hordein genes in barley endosperm is inversely correlated with gene activity and affected by the regulatory gene *Lys3*. *Proceedings of the National Academy of Sciences of the United States of America*.

[45] Nielsen ME, Lok F, Nielsen HB (2006). Distinct developmental defense activations in barley embryos identified by transcriptome profiling. *Plant Molecular Biology*.

[46] Potokina E, Caspers M, Prasad M (2004). Functional association between malting quality trait components and cDNA array based expression patterns in barley (*Hordeum vulgare* L.). *Molecular Breeding*.

[47] Potokina E, Druka A, Luo Z, Wise R, Waugh R, Kearsey MJ (2008). Gene expression quantitative trait locus analysis of 16 000 barley genes reveals a complex pattern of genome-wide transcriptional regulation. *The Plant Journal*.

[48] Waugh R, Leader DJ, McCallum N, Caldwell D (2006). Harvesting the potential of induced biological diversity. *Trends in Plant Science*.

[49] Caldwell DG, McCallum N, Shaw P, Muehlbauer GJ, Marshall DF, Waugh R (2004). A structured mutant population for forward and reverse genetics in Barley (*Hordeum vulgare* L.). *The Plant Journal*.

[50] Koprek T, McElroy D, Louwerse J, Williams-Carrier R, Lemaux PG (2000). An efficient method for dispersing *Ds* elements in the barley genome as a tool for determining gene function. *The Plant Journal*.

[51] Cooper LD, Marquez-Cedillo L, Singh J (2004). Mapping *Ds* insertions in barley using a sequence-based approach. *Molecular Genetics and Genomics*.

[52] Singh J, Zhang S, Chen C (2006). High-frequency *Ds* remobilization over multiple generations in barley facilitates gene tagging in large genome cereals. *Plant Molecular Biology*.

[53] Zhao T, Palotta M, Langridge P (2006). Mapped *Ds*/T-DNA launch pads for functional genomics in barley. *The Plant Journal*.

[54] Ayliffe MA, Pallotta M, Langridge P, Pryor AJ (2007). A barley activation tagging system. *Plant Molecular Biology*.

[55] Forster BP, Franckowiak JD, Lundqvist U, Lyon J, Pitkethly I, Thomas WTB (2007). The barley phytomer. *Annals of Botany*.

[56] Qu S, Desai A, Wing R, Sundaresan V (2008). A versatile transposon-based activation tag vector system for functional genomics in cereals and other monocot plants. *Plant Physiology*.

[57] Holm PB, Olsen O, Schnorf M, Brinch-Pedersen H, Knudsen S (2000). Transformation of barley by microinjection into isolated zygote protoplasts. *Transgenic Research*.

[58] Hensel G, Valkov V, Middlefell-Williams J, Kumlehn J (2008). Efficient generation of transgenic barley: the way forward to modulate plant-microbe interactions. *Journal of Plant Physiology*.

[59] Kumlehn J, Serazetdinova L, Hensel G, Becker D, Loerz H (2006). Genetic transformation of barley (*Hordeum vulgare* L.) via infection of androgenetic pollen cultures with *Agrobacterium tumefaciens*. *Plant Biotechnology Journal*.

[60] von Wettstein D (2007). From analysis of mutants to genetic engineering. *Annual Review of Plant Biology*.

[61] Kihara M, Okada Y, Kuroda H, Saeki K, Yoshigi N, Ito K (2000). Improvement of 
*β*-amylase thermostability in transgenic barley seeds and transgene stability in progeny. *Molecular Breeding*.

[62] Scheidig A, Fröhlich A, Schulze S, Lloyd JR, Kossmann J (2002). Downregulation of a chloroplast-targeted 
*β*-amylase leads to a starch-excess phenotype in leaves. *The Plant Journal*.

[63] Souppe J, Beudeker RF (2002). Process for the production of alcoholic beverages using MaltSeed.

[64] Jensen LG, Olsen O, Kops O, Wolf N, Thomsen KK, von Wettstein D (1996). Transgenic barley expressing a protein-engineered, thermostable (1,3-1,4)-
*β*-glucanase during germination. *Proceedings of the National Academy of Sciences of the United States of America*.

[65] Horvath H, Huang J, Wong O (2000). The production of recombinant proteins in transgenic barley grains. *Proceedings of the National Academy of Sciences of the United States of America*.

[66] Nuutila AM, Ritala A, Skadsen RW, Mannonen L, Kauppinen V (1999). Expression of fungal thermotolerant endo-1,4-
*β*-glucanase in transgenic barley seeds during germination. *Plant Molecular Biology*.

[67] von Wettstein D, Mikhaylenko G, Froseth JA, Kannangara CG (2000). Improved barley broiler feed with transgenic malt containing heat-stable (1,3-1,4)-
*β*-glucanase. *Proceedings of the National Academy of Sciences of the United States of America*.

[68] von Wettstein D, Warner J, Kannangara GG (2003). Supplements of transgenic malt or grain containing (1,3-1,4)-
*β*-glucanase increase the nutritive value of barley-based broiler diets to that of maize. *British Poultry Science*.

[69] Xue GP, Patel M, Johnson JS, Smyth DJ, Vickers CE (2003). Selectable marker-free transgenic barley producing a high level of cellulase (1,4-
*β*-glucanase) in developing grains. *Plant Cell Reports*.

[70] Stahl Y, Coates S, Bryce JH, Morris PC (2004). Antisense downregulation of the barley limit dextrinase inhibitor modulates starch granule size distribution, starch composition and amylopectin structure. *The Plant Journal*.

[71] Cho M-J, Wong JH, Marx C, Jiang W, Lemaux PG, Buchanan BB (1999). Overexpression of thioredoxin *h* leads to enhanced activity of starch debranching enzyme (pullulanase) in barley grain. *Proceedings of the National Academy of Sciences of the United States of America*.

[72] Wong JH, Kim Y-B, Ren P-H (2002). Transgenic barley grain overexpressing thioredoxin shows evidence that the starchy endosperm communicates with the embryo and the aleurone. *Proceedings of the National Academy of Sciences of the United States of America*.

[73] Sun C, Höglund A-S, Olsson H, Mangelsen E, Jansson C (2005). Antisense oligodeoxynucleotide inhibition as a potent strategy in plant biology: identification of SUSIBA2 as a transcriptional activator in plant sugar signalling. *The Plant Journal*.

[74] Sun C, Ridderstråle K, Höglund A-S, Larsson L-G, Jansson C (2007). Sweet delivery—sugar translocators as ports of entry for antisense oligodeoxynucleotides in plant cells. *The Plant Journal*.

[75] Murray F, Kalla R, Jacobsen J, Gubler F (2003). A role for HvGAMYB in anther development. *The Plant Journal*.

[76] Sreenivasulu N, Altschmied L, Panitz R (2001). Identification of genes specifically expressed in maternal and filial tissues of barley
caryopses: a cDNA array analysis. *Molecular Genetics and Genomics*.

[77] Radchuk V, Borisjuk L, Radchuk R (2006). Jekyll encodes a novel protein involved in the sexual reproduction of barley. *Plant Cell*.

[78] Künne C, Lange M, Funke T (2005). CR-EST: a resource for crop ESTs. *Nucleic Acids Research*.

[79] Weise S, Grosse I, Klukas C (2006). Meta-All: a system for managing metabolic pathway information. *BMC Bioinformatics*.

[80] Grafahrend-Belau E, Weise S, Koschützki D, Scholz U, Junker BH, Schreiber F (2008). MetaCrop: a detailed database of crop plant metabolism. *Nucleic Acids Research*.

[81] Junker BH, Klukas C, Schreiber F (2006). VANTED: a system for advanced data analysis and visualization in the context of biological networks. *BMC Bioinformatics*.

[82] Rolletschek H, Weschke W, Weber H, Wobus U, Borisjuk L (2004). Energy state and its control on seed development: starch accumulation is associated with high ATP and steep oxygen gradients within barley grains. *Journal of Experimental Botany*.

[83] Gubatz S, Dercksen VJ, Brüß C, Weschke W, Wobus U (2007). Analysis of barley (*Hordeum vulgare*) grain development using three-dimensional digital models. *The Plant Journal*.

[84] Stark M, Manz B, Ehlers A (2007). Multiparametric high-resolution imaging of barley embryos by multiphoton microscopy and magnetic resonance micro-imaging. *Microscopy Research and Technique*.

[85] Neuberger T, Sreenivasulu N, Rokitta M (2008). Quantitative imaging of oil storage in developing crop seeds. *Plant Biotechnology Journal*.

[86] Junker BH, Koschützki D, Schreiber F (2006). Kinetic modeling with the systems biology modelling environment SyBME. *Journal of Integrative Bioinformatics*.

[87] Koch I, Junker BH, Heiner M (2005). Application of Petri net theory for modelling and validation of the sucrose breakdown pathway in the potato tuber. *Bioinformatics*.

[88] Finnie C, Melchior S, Roepstorff P, Svensson B (2002). Proteome analysis of grain filling and seed maturation in barley. *Plant Physiology*.

[89] Witzel K, Surabhi G-K, Jyothsnakumari G, Sudhakar C, Matros A, Mock H-P (2007). Quantitative proteome analysis of barley seeds using
ruthenium(II)-tris-(bathophenanthroline-disulphonate) staining. *Journal of Proteome Research*.

[90] Graner A, Jahoor A, Schondelmaier J (1991). Construction of an RFLP map of barley. *Theoretical and Applied Genetics*.

[91] Heun M, Kennedy AE, Anderson JA, Lapitan NLV, Sorrells ME, Tanksley SD (1991). Construction of a restriction fragment length polymorphism map for
barley (*Hordeum vulgare*). *Genome*.

[92] Kleinhofs A, Kilian A, Saghai Maroof MA (1993). A molecular, isozyme and morphological map of the barley (*Hordeum vulgare*) genome. *Theoretical and Applied Genetics*.

[93] Hayes PM, Castro A, Marquez-Cedillo L, von Bothmer R, van Hintum T, Knüpffer H, Sato K (2003). Genetic diversity for quantitatively inherited agronomic and malting quality traits. *Diversity in Barley (*Hordeum vulgare*)*.

[94] Friedt W, Ordon F, Varshney R, Tuberosa R (2008). Molecular markers for gene pyramiding and resistance breeding in barley. *Genomics-Assisted Crop Improvement, Vol 2: Genomics Applications in Crops*.

[95] Thiel T, Michalek W, Varshney RK, Graner A (2003). Exploiting EST databases for the development and characterization of gene-derived SSR-markers in barley (*Hordeum vulgare* L.). *Theoretical and Applied Genetics*.

[96] Kota R, Wolf M, Michalek W, Graner A (2001). Application of denaturing high-performance liquid chromatography for mapping of single nucleotide polymorphisms in barley (*Hordeum vulgare* L.). *Genome*.

[97] Kota R, Varshney RK, Prasad M, Zhang H, Stein N, Graner A EST-derived single nucleotide polymorphism markers for assembling genetic and
physical maps of the barley genome.

[98] Kota R, Rudd S, Facius A (2003). Snipping polymorphisms from large EST collections in barley (*Hordeum vulgare* L.). *Molecular Genetics and Genomics*.

[99] Thiel T, Kota R, Grosse I, Stein N, Graner A (2004). SNP2CAPS: a SNP and INDEL analysis tool for CAPS marker development. *Nucleic Acids Research*.

[100] Karakousis A, Gustafson JP, Chalmers KJ, Barr AR, Langridge P (2003). A consensus map of barley integrating SSR, RFLP, and AFLP markers. *Australian Journal of Agricultural Research*.

[101] Diab AA (2006). Construction of barley consensus map showing chromosomal regions associated with economically important traits. *African Journal of Biotechnology*.

[102] Varshney RK, Marcel TC, Ramsay L (2007). A high density barley microsatellite consensus map with 775 SSR loci. *Theoretical and Applied Genetics*.

[103] Rostoks N, Ramsay L, MacKenzie K (2006). Recent history of artificial outcrossing facilitates whole-genome association mapping in elite inbred crop varietes. *Proceedings of the National Academy of Sciences of the United States of America*.

[104] Wenzl P, Carling J, Kudrna D (2004). Diversity Arrays Technology (DArT) for whole-genome profiling of barley. *Proceedings of the National Academy of Sciences of the United States of America*.

[105] Wenzl P, Li H, Carling J (2006). A high-density consensus map of barley linking DArT markers to SSR, RFLP and STS loci and agricultural traits. *BMC Genomics*.

[106] Rostoks N, Borevitz JO, Hedley PE (2005). Single-feature polymorphism discovery in the barley transcriptome. *Genome Biology*.

[107] Graner A, Bauer E (1993). RFLP mapping of the rym4 virus resistance gene in barley. *Theoretical and Applied Genetics*.

[108] Graner A, Streng S, Kellermann A (1999). Molecular mapping and genetic fine-structure of the rym5 locus encoding
resistance to different strains of the barley yellow mosaic virus complex. *Theoretical and Applied Genetics*.

[109] Stein N, Perovic D, Kumlehn J (2005). The eukaryotic translation initiation factor 4E confers multiallelic recessive
Bymovirus resistance in *Hordeum vulgare* (L.). *The Plant Journal*.

[110] Mammadov JA, Brooks WS, Griffey CA, Saghai Maroof MA (2007). Validating molecular markers for barley leaf rust resistance genes Rph5 and Rph7. *Plant Breeding*.

[111] Werner K, Friedt W, Ordon F (2005). Strategies for pyramiding resistance genes against the barley yellow mosaic
virus complex (BaMMV, BaYMV, BaYMV-2). *Molecular Breeding*.

[112] Salvi S, Tuberosa R (2005). To clone or not to clone plant QTLs: present and future challenges. *Trends in Plant Science*.

[113] Sutton T, Baumann U, Hayes J (2007). Boron-toxicity tolerance in barley arising from efflux transporter amplification. *Science*.

[114] Rae SJ, Macaulay M, Ramsay L (2007). Molecular barley breeding. *Euphytica*.

[115] Thomas WTB (2003). Prospects for molecular breeding of barley. *Annals of Applied Biology*.

[116] Doss S, Schadt EE, Drake TA, Lusis AJ (2005). *Cis*-acting expression quantitative trait loci in mice. *Genome Research*.

[117] Reich DE, Cargill M, Bolk S (2001). Linkage disequilibrium in the human genome. *Nature*.

[118] Rafalski A, Morgante M (2004). Corn and humans: recombination and linkage disequilibrium in two genomes of similar size. *Trends in Genetics*.

[119] Gupta PK, Rustgi S, Kulwal PL (2005). Linkage disequilibrium and association studies in higher plants: present status and future prospects. *Plant Molecular Biology*.

[120] Kraakman ATW, Niks RE, Van den Berg PM, Stam P, Van Eeuwijk FA (2004). Linkage disequilibrium mapping of yield and yield stability in modern spring barley cultivars. *Genetics*.

[121] Romagosa I, Han F, Ullrich SE, Hayes PM, Wesenberg DM (1999). Verification of yield QTL through realized molecular marker-assisted selection responses in a barley cross. *Molecular Breeding*.

[122] Li JZ, Huang XQ, Heinrichs F, Ganal MW, Röder MS (2006). Analysis of QTLs for yield components, agronomic traits, and disease resistance in an advanced backcross population of spring barley. *Genome*.

[123] Malysheva-Otto LV, Ganal MW, Röder MS (2006). Analysis of molecular diversity, population structure and linkage disequilibrium
in a worldwide survey of cultivated barley germplasm (*Hordeum vulgare* L.). *BMC Genetics*.

[124] Caldwell KS, Russell J, Langridge P, Powell W (2006). Extreme population-dependent linkage disequilibrium detected in an
inbreeding plant species, *Hordeum vulgare*. *Genetics*.

[125] Morrell PL, Toleno DM, Lundy KE, Clegg MT (2005). Low levels of linkage disequilibrium in wild barley (*Hordeum vulgare* ssp. *spontaneum*) despite high rates of self-fertilization. *Proceedings of the National Academy of Sciences of the United States of America*.

[126] Steffenson BJ, Olivera P, Roy JK, Jin Y, Smith KP, Muehlbauer GJ (2007). A walk on the wild side: mining wild wheat and barley collections for rust resistance genes. *Australian Journal of Agricultural Research*.

[127] Stracke S, Presterl T, Stein N, Perovic D, Ordon F, Graner A (2007). Effects of introgression and recombination on haplotype
structure and linkage disequilibrium surrounding a locus encoding Bymovirus resistance in barley. *Genetics*.

[128] Jansen RC, Nap J-P (2001). Genetical genomics: the added value from segregation. *Trends in Genetics*.

[129] Sreenivasulu N, Varshney RK, Kavi-Kishor PB, Weschke W, Gupta PK, Varshney RK (2004). Functional genomics for tolerance to abiotic stress in cereals. *Cereal Genomics*.

[130] Varshney RK, Graner A, Sorrells ME (2005). Genomics-assisted breeding for crop improvement. *Trends in Plant Science*.

[131] Potokina E, Prasad M, Malysheva L, Röder MS, Graner A (2006). Expression genetics and haplotype analysis reveal *cis*
regulation of serine carboxypeptidase I (Cxp1), a candidate gene for malting quality in barley (*Hordeum vulgare* L.). *Functional and Integrative Genomics*.

[132] Li JZ, Huang XQ, Heinrichs F, Ganal MW, Röder MS (2005). Analysis of QTLs for yield, yield components, and malting quality in a BC3-DH population of spring barley. *Theoretical and Applied Genetics*.

[133] Wicker T, Schlagenhauf E, Graner A, Close TJ, Keller B, Stein N (2006). 454 sequencing put to the test using the complex genome of barley. *BMC Genomics*.

[134] Hammer G, Cooper M, Tardieu F (2006). Models for navigating biological complexity in breeding improved crop plants. *Trends in Plant Science*.

[135] Yin X, Struik PC, Kropff MJ (2004). Role of crop physiology in predicting gene-to-phenotype relationships. *Trends in Plant Science*.

[136] Langridge P, Tuberosa R, Phillips RL, Gale M (2005). Molecular breeding of wheat and barley. *The Wake of Double Helix: From the Green Revolution to the Gene Revolution*.

